# 3D Image-Guided Automatic Pipette Positioning for Single Cell Experiments *in vivo*

**DOI:** 10.1038/srep18426

**Published:** 2015-12-22

**Authors:** Brian Long, Lu Li, Ulf Knoblich, Hongkui Zeng, Hanchuan Peng

**Affiliations:** 1 Allen Institute for Brain Science, Seattle, WA, USA

## Abstract

We report a method to facilitate single cell, image-guided experiments including *in vivo* electrophysiology and electroporation. Our method combines 3D image data acquisition, visualization and on-line image analysis with precise control of physical probes such as electrophysiology microelectrodes in brain tissue *in vivo*. Adaptive pipette positioning provides a platform for future advances in automated, single cell *in vivo* experiments.

Observing and understanding the roles of individual cells in live tissue is a substantial challenge across biology, especially in neuroscience where each neuron’s activity is intimately coupled to the state and dynamics of other connected cells. New tools are needed to access individual cells for a variety of experiments, especially in the context of high-throughput studies of cellular physiology. Here we use automated pipette positioning to facilitate access to single neurons for *in vivo* experiments using two-photon fluorescence microscopy. Our method takes advantage of genetically-encoded fluorescent labeling to target various cell types in the mouse cortex and approach the target cell with a pipette to provide physical access for measurement and perturbation of single cells^1^,^2^. Physical access to the targeted cell with a pipette is integral to three important experimental modalities: First, electrophysiological measurements from single cells provide unparalleled resolution of cellular electrical activity^3^. Second, physical access to targeted cells provides a way to extract cellular contents for subsequent biochemical analysis. Lastly, single-cell electroporation and microinjection can deliver substances into the cell including dyes for morphological characterization and plasmids to drive expression of exogenous genes^4^,^5^.

Targeting a physical probe to specific cell types in the mouse neocortex is usually done either manually with two-photon-based visual guidance^6^ or ‘blind’^3^ based solely on electrical measurements and depth below the pia. Blind electrophysiology experiments in the brain, including automated methods^7^,^8^, are strongly biased towards recording from larger, more robust cell types such as pyramidal neurons; to achieve cell-type specificity requires targeted experiments with visual guidance. Manually guiding a pipette to a targeted cell deep in tissue presents several practical difficulties that make these experiments challenging. First, the process of bringing a pipette in close proximity to a target cell requires close attention and extensive human expertise to control micromanipulators with visual feedback as the pipette moves through the tissue. Second, this positioning process requires precision and stability to minimize lateral (off-axial) movements near the target cell that can cause mechanical deformation and disruption of the target and neighboring cells. Third, efficiency and robustness of the approach process is critical because an excessive number of insertions can lead to inflammation and edema, conditions under which no reliable measurements can be made. Finally, *in vivo* measurements such as cell-attached and whole-cell electrophysiology recordings are highly sensitive to the condition of the physical probe at the membrane of the target cell. The conventional process of approaching a targeted cell typically involves careful manual control of both the pipette micromanipulator and the microscope objective, as well as visual monitoring of pipette resistance and adjustment of the fluid flow from the pipette by manual pressure control. This highly specialized process requires months to years of training and practice to become proficient.

This challenging experimental paradigm provides a test case for applying 3D image analysis and computerized probe control to assist *in vivo* experiments. In this context, we have developed a system called smartACT (smart, Adaptive Cell Targeting) that uses volumetric image information to adaptively approach a user-targeted cell in 3D (Fig. 1). We demonstrate incorporation of volumetric image information and pipette automation into a suite of 2-P targeted experiments in the mouse brain. The smartACT system improves two critical phases common to several probe-based, 2-P-targeted experimental techniques: first, choosing a target cell and identifying its 3D location, and second, bringing the pipette in close proximity to the target cell. By adaptively planning and executing a pipette trajectory based on 3D image data, our system is able to approach targeted cells automatically in a comparable time to a human operator. Future studies exploring cellular function *in vivo* in a systematic manner will benefit from automation for targeting measurements and perturbations at the single-cell level.

With the smartACT system, we collect 3D image data of fluorescently-labeled neurons in the mouse cortex using *in vivo* 2-P imaging (Fig. 1A), visualize the probe tip and target neurons and plan a trajectory for probe movement as illustrated in Fig. 1B. The smartACT workflow (Fig. 1D, Supplemental Movie M1) includes collecting a scan containing the pipette tip and the neurons of interest. We then display 3D volumetric image data in Vaa3D^9^, which allows rapid 3D visualization of the brain and fluorescently labeled neurons in the neocortex, limited only by the depth of 2-P scanning microscopy (Fig. 1C). 3D visualization, especially the ability to rotate and view the data in three dimensions, lets the user quickly grasp spatial relationships in the data that are important for target selection. Our method of 3D target selection includes single computer-mouse operation (e.g. one click) ‘Virtual-Finger’ technology^10^ and gives the user an intuitive and very efficient interface for precisely locating the tip of the pipette and the center of the target cell. In our configuration with (1.23 × 1.23 × 2) μm voxel size, this target selection method is precise (mean square deviation = 2.32 μm from N = 25 localizations) [Fig S1] and yields accurate localization of the pipette tip and target cell within the image.

Having localized the pipette tip and target neuron in 3D, we first tested a naïve approach. This was done by calculating the path to the cell that approaches along the pipette axis and terminates at a user-specified distance (the ‘buffer distance’) from the center of the target cell. The path within the cortex is subdivided into a sequence of discrete axial movements (steps) whose size is user-defined; 2–4 μm steps and 10–12 μm buffer distance were used in the data presented here. The naïve approach, which is similar to manual techniques, positions the pipette tip in the vicinity of the target, with the final lateral distance (closest distance from the target to the pipette axis) *r*_*lateral*_ = 12.2 ± 7.1 μm (all values reported are mean ± standard deviation, N = 22, see Fig. S2). This final displacement results from contributions of 9.7 ± 7.1 μm and 6.0 ± 3.2 μm lateral displacements from pipette deflection and target cell displacement, respectively. This range of final positions may be traditionally accepted as sufficiently precise for manual 2-P targeted electrophysiology, however it is problematically inaccurate for automated experiments. For targeting a mouse cortical pyramidal neuron with a 10 μm-diameter, the error in this naïve approach is comparable to the size of the cell body. Specifically, the final lateral distance between the cell and the pipette axis requires substantial manual adjustment to reach the target location on the surface of the cell; all while the pipette is deep in the cortex.

In response to this wide variability in final pipette positions relative to the target cell, we developed an adaptive method that takes advantage of volumetric image data collected at an intermediate point along the route to the target. In the adaptive step, smartACT collects a new 3D image substack that scans the z range of the pipette tip and target, automatically locates the tip and target within the substack, and adapts the initial trajectory to compensate for the displacement of the pipette and target, improving the accuracy of cell targeting.

To assess the performance of our adaptive method, we first performed tests *in vitro.* The *in vitro* tests consisted of targeting 2 μm fluorescent beads suspended in agarose gel. Instead of choosing the bead center as the target, we choose a target location several microns from the bead and used the adaptive positioning to determine the correct approach to the target. SmartACT was able to correct the intentional targeting error to a large extent, reducing the final lateral distance from *r*_*lateral*_ = 7.29 ± 2.99 μm at the adaptive step to *r*_*lateral*_ = 3.04 ± 2.00 μm at the final position ~12 μm (the buffer distance) from the target (N = 18) (Fig. 1E,F, S3). We next tested the adaptive method to target fluorescently-labeled neurons in mouse primary visual cortex (V1) *in vivo*. To approach targeted V1 neurons, we chose relatively low pixel resolution 2-P image stacks (256 × 256 × *N*, where *N* is ca. 150 to include pipette tip and target cell) to increase fluorescence signal to noise ratio (SNR), improve imaging speed and reduce photodamage. Results show smartACT’s image-based adaptive positioning leads to dramatic correction of deviations measured at the adaptive step and substantial improvement over non-adaptive approaches [Fig S2 – S4]. At the end of the approach *in vivo*, smartACT achieves good alignment of the pipette axis to the target cell body (*r*_*lateral*_ = 5.04 ± 2.93 μm, N = 11), which is less than half of the average lateral distance measured at the adaptive correction step. Since a typical mouse neuron’s cell body radius is about 5 μm, the final distance from the pipette to the cell surface is 12.33 ± 7.99 μm, indicating that adaptive pipette movements can target the pipette to single neurons *in vivo* (Fig. 1E,F, S3, S4). By adopting the adaptive approach, smartACT reduces the lateral (off-axis) displacement and refines the position of the pipette relative to the target.

This adaptive step requires sufficient SNR and image quality to segment both the pipette tip and target cell, so smartACT utilizes signals from different fluorophores to enhance image contrast and SNR. We tested this functionality in a transgenic mouse line Cux2-CreERT2;Ai14 (“Cux2”), in which layer (L) 2/3 excitatory pyramidal neurons are densely labeled with the cytosolic red fluorescent protein tdTomato. With the pipette filled with a green fluorescent dye (Alexa 488), we visualized pipette tip and labeled neurons in green and red channels, respectively, and smartACT successfully localized pipette tip and targeted neurons and brought the pipette to the targeted cell precisely (*r*_*lateral*_ = 4.04 ± 2.59 μm, N = 11 approaches in 3 mice even in the high background fluorescence signal from dense soma and extensively labeled dendritic arborizations. This high degree of accuracy is achieved without sacrificing experiment efficiency: our method requires comparable or less time than a manual approach, with the entire adaptive approach process (Steps 2–10 in Fig. 1D) taking 6:55 ± 0:53 min:sec, including approximately 2:30 for image acquisition (Fig. S5). In three manual experiments, an expert (L.L.) was timed to take 3–4 minutes to go from the initial pipette position above the brain to a position near the targeted cell, and during this time, the brain was continually imaged. Although our current implementation devotes over 2 minutes to image acquisitions (which could be reduced with faster imaging modalities such as resonant scanning), there are clear benefits to acquiring image data during the approach process. In particular, smartACT reduces the total imaging time during the approach and includes images of a full 3D scan of the target field of view from above the surface of the brain to the target depth as well as images and quantification of pipette and cell locations before and during the approach to the target. This image data may be especially useful for monitoring cell locations and brain health over long-term experiments or quantifying tissue deformation during pipette approaches.

The high degree of accuracy of our final pipette approach provides the user with a repeatable starting position for manual fine adjustments to begin electrophysiology or electroporation experiments. To demonstrate the utility of smartACT for *in vivo* research, we applied it to three different experiments using two-photon imaging and a glass pipette to access individual cells in the anesthetized mouse V1. Experiments included whole-cell recordings and cell-attached recordings from genetically defined cell types in response to visual stimulations and single-cell electroporation to drive EGFP expression in targeted V1 neurons (see Methods). To illustrate the general applicability of smartACT to multiple cell types, in separate experiments we approached large, excitatory pyramidal cells (Cux2) and small, inhibitory interneurons (SST-IRES-Cre;Ai14, “SST”) [Fig. 2, S6].

In these experiments, the accessible region of the brain is small (~300–500 microns in x-y) because of physical constraints (objective shape and position, pipette angle, skull and headplate position) and the need to minimize the size of the craniotomy and durotomy. The small accessible region makes accurate approaches critical, and 3D visualization allows the user to easily see the spatial relationship between target cells and other features such as the borders of the craniotomy. At the termination of the adaptive approach, the pipette tip was positioned in close proximity to the targeted cells (lateral displacement *r*_*lateral*_ = 4.2 ± 2.7 μm and axial distance from cell center *r*_*axial*_ = 16.1 ± 7.1 μm for N = 15 approaches from these experiments). From the end of the smartACT trajectory we used manual manipulator control to place the pipette tip against the target neuron and whole-cell recording can be readily achieved (4 out of 6 approaches in N = 2 Cux2 mice and 2 whole-cell and 3 cell-attached recordings out of 9 approaches in N = 2 SST mice) with standard techniques^6^. The overall success rate (9 recordings out of 17 attempts in four animals) was comparable to our experience with a completely manual approach. Failure to achieve a recording was typically due to problems with seal formation or break-in while attempting whole-cell configuration, and were not noticeably more frequent than manual approaches by an expert (L.L.). 2-P imaging and pipette movement mediated by smartACT had no discernable negative impact on the membrane properties of the target neurons. In particular, recorded neurons show normal electrophysiological properties such as resting membrane potential (Vm), over-shooting action potentials, Vm transition between up- and down-states and visually evoked responses, consistent with published work (Fig. 2B,D).

Single-cell electroporation of genetically defined neurons can provide gene delivery to individual neurons of a targeted cell type. This individual neuron can then be imaged at a later time point and interrogated optically or electrophysiologically^11^,^12^,^13^,^14^. We utilized smartACT to approach the target cell and after a final manual adjustment to bring the pipette in contact with the cell, we applied a train of current pulses, disrupting the plasma membrane of the target cell^4^,^6^. Influx of pipette dye (Alexa 488 in our case) into the target neuron after applying the current train indicates successful delivery of extraneous material into the target neuron, while the retained tdTomato fluorescence shows that the membrane disruption was temporary, as cytosolic material did not leave the cell (Fig 2E). Application of our method to single-cell electroporation confirms that, in addition to accessing single-cell physiology measurements, smartACT can facilitate perturbations of an individual target cell.

We have created an automated system to facilitate targeted experiments in single cells by using on-line, volumetric image information to adaptively control pipette movement in the brain. Our method results in lower variability than automatic approaches without adaptive image information and may simplify 2-P-targeted experiments for novice users by reducing the amount of manual manipulator control. In our experience, the smartACT system has been helpful to an experienced manual patcher (L.L.) by reducing the attentional load and manual involvement during target cell approach. Additionally, when the time for image collection is considered, the time required for our method is comparable to a human operator, generating image data and experimental parameters such as pipette and target position available for on-line and post-hoc analysis. Image-guided adaptive pipette movement may also be a useful addition for automated and semi-automated electrophysiology *in vitro*^7^,^8^,^15^,^16^. In future studies, our principal method of using adaptive pipette movement could be applied to more challenging experimental paradigms, such as fine-tuning the pipette position and completing electrophysiological recordings or compensating for movement of targeted neurons in awake, behaving animals. Our results establish the feasibility of using image data for adaptive pipette control *in vivo* and indicate that smartACT is a viable first step towards completely automated, targeted electrophysiology experiments in the brain.

## Methods

Experimental procedures pertaining to the use of mice were conducted according to NIH guidelines and were approved by the Institutional Animal Care and Use Committee (IACUC) of the Allen Institute for Brain Science.

### Animals

To visualize and target single neurons in the brain, transgenic mice expressing a red fluorescent protein tdTomato) under the control of promoters of defined neurochemical markers were used. Adaptive approach experiments were performed in two lines: Cux2-CreERT2;Ai14, which has a dense labeling of excitatory pyramidal neuron in layer 2/3 (n = 3), and Sst-IRES-Cre;Ai14, which labels Somatostatin (SST) expressing inhibitory interneurons (n = 3).

### Surgical procedures

Briefly, adult mice were implanted with a metal headplate. After at least 3 days of recovery, mice were anesthetized first with Ketamine/Xylazine mixture and then Isoflurane (0.75–1.5% in O_2_). Body temperature was monitored and maintained at 37 C with a feedback controlled animal heating pad (Harvard Apparatus, USA). A circular craniotomy (~1.5 mm in diameter) was made over the right primary visual cortex (V1) centering on 1.25 mm anterior and 2.25 mm lateral to the Lambda. To facilitate the penetration of the probe (a glass pipette in our cases), a small durotomy was carefully done to expose the region of interest in V1 avoiding major blood vessels. Then the craniotomy was covered with a thin layer of low melting-point agarose (Sigma-Aldrich, USA) to reduce the motion in living brain. The mouse was transferred to the imaging setup for 2-p imaging. During the entire course of imaging/recording, animals were kept warm and appropriately anesthetized.

### 2-p Imaging and Probe Positioning

2-p imaging was performed using a Moveable Objective Microscope (MOM, Sutter, CA USA) and a tunable Chameleon Ultra II laser (Coherent USA). The MOM was coupled with a 40× water-immersion objective (LUMPLFLN 40XW, Olympus USA), controlled by the open-source software ScanImage 3.8 (Janelia Research Campus/ Vidrio Technologies). Briefly, a borosilicate microelectrode (glass pipette) was installed on a MultiClamp 700B headstage (Molecular Devices, USA) mounted onto a MP-285 4-axis manipulator (Sutter USA). The headstage was positioned so the pipette pointed anterior, oriented approximately 31 degrees down from horizontal. The tip of the glass pipette was first visualized under widefield imaging with a color CCD camera (A-M Scope, WA) and manually positioned ~50–200 μm over the durotomy region. This was done by first coarsely moving the pipette tip into the field-of-view of a 4× objective (Olympus USA) then refining its position under the 40× objective. Under 4× and 40× magnification, the target area was chosen to avoid visible surface vasculature in both the target area and the adjacent posterior region where the pipette would enter the cortex. We chose the excitation wavelength at 920 nm to visualize both the target neuron with red cytosolic fluorophore tdTomato and glass pipette containing a fluorescent dye, Alexa-488, for instance. Typically 256X256 frame-size image-stacks were acquired by ScanImage, containing a short segment of the pipette tip, down to a depth in the cortex specified by the experimenter. On the completion of imaging, image stack was automatically processed by the smartACT program: the location of pipette tip and soma of neuron to-be-targeted was determined through interactive procedures between the smartACT program and experimenter, then the trajectory of pipette movement was computed based on the approaching angle of the pipette (31^o^ in our case) and approaching initiated (see below). To reduce photodamage during multiple imaging/targeting processes, laser power at the sample plane was below a maximum value of 75 mW, with typical power used for z stacks at 10–50% of that value.

### Software Control and Automation

Analysis and automation software included Vaa3D (www.vaa3d.org), and custom analysis routines and user interfaces written in MATLAB, utilizing the microscope control and MP-285 driver available in ScanImage 3.8 (Vidrio Technologies http://vidriotechnologies.com/). The source code and user instructions for the custom analysis routines and user interfaces are available as a supplemental data file under the GPLv3 open software license^17^. All reported values are mean ± standard deviation. The standard deviation (as opposed to standard error of the mean) was reported to clearly quantify the distribution of pipette-target distances during use of smartACT.

#### Initial targeting

Initial localization of pipette tip, target cell and pial surface was done in Vaa3D using single-click virtual finger technology. The planned trajectory included a retraction step up and away from the brain surface, translation to the entry location (located along a vector parallel to the pipette axis and intersecting the target cell) and termination at a point *R* distance from the target location along the pipette access. The distance R (typically set at 10–12 microns) is the target buffer distance, supplied in by the user in the interface.

#### Automatic pipette tip and target cell localization

First, a new z stack (substack) was collected including the expected pipette tip and target cell locations. From this substack, 3D regions of interest (ROI) around the expected pipette tip and cell locations were extracted and the actual tip and cell locations measured.

#### Pipette tip localization

Within the substack around the pipette, the minimum intensity is subtracted from all pixels and the intensity values are then normalized to the range 5th percentile – *p*^th^ percentile, where *p* is user-adjustable and defaults to 98th percentile. This normalized 3D image is then smoothed in 3D with a 3 × 3 × 1 boxcar filter, and maximum intensity projections (MIPs) are created along each dimension. These 3 MIP images are segmented by thresholding at a user-adjustable value (default at 90% of image max). The tip is located within these 3 segmented images by choosing the largest object with greater than 10 pixels. We extract the 3D tip location from the three projections of the tip object by first selecting the 10 pixels at the tip in each projection, and then identifying the ‘deepest’ pixel (z) location, the most anterior pixel (x) location from the x-z image and the average pixel locations from the x-y image. Importantly, at this stage in the approach process, the pressure in the pipette is low, both for the purposes of pipette localization and to reduce the amount of pipette internal solution in the brain.

#### Target localization

Within the substack around the nominal cell body location, the image data is filtered with a 3D Gaussian bandpass filter with two characteristic sizes (2 and 20 pixels lower and upper limits of the pass band) to highlight round features roughly 20 pixels in size. The resulting image is segmented into putative cells by thresholding at a user-adjustable value (default at 90% of image max), and small objects (less than 10 voxels) are eliminated. If there is more than one target remaining in the segmented image, the target object is identified as the object whose center of mass is closest to the original target location.

#### Adaptive pipette positioning

Following tip and target localization, the pipette trajectory was modified by first calculating the lateral component of the pipette tip offset and compensating for that lateral deflection, then translating the resulting approach path by the displacement of the target to compensate for target cell movement. Subsequent steps by the manipulator were automatically executed along this modified trajectory.

#### Assessing precision of single-click targeting

Two *in vivo* image volumes and one *in vitro* image volume (a dilute suspension of 2 μm fluorescent beads in 1.2% low-melt agarose) were used to assess the precision of single-click targeting using Vaa3D. Each pipette tip or target was clicked on from a wide range of angles, creating an independent localization attempt for each click. Lateral and axial components of the pipette tip locations in Fig. S1 were measured from the line parallel to the nominal pipette axis, through the mean of all pipette localizations.

#### Post-hoc assessment of pipette-cell separation

To quantify the precision of smartACT approaches, pipette tip and target cell locations were measured manually. For all analyzed approaches (N = 18 *in vitro*, N = 11 *in vivo* approaches for method characterization, N = 15 *in vivo* approaches for electrophysiology and electroporation experiments), pipette tip and target location were identified using single- or two-click, virtual-finger methods in Vaa3D. Out of the 26 total *in vivo* approaches, one outlier approach with very low 2-p signal to noise ratio resulted in inaccurate pipette localization (final position 45.8 μm from target cell) and was excluded from subsequent analysis. Lateral and axial components of the final pipette tip locations were measured from the line parallel to the nominal pipette axis through the final cell location.

### 2-p Targeted Electrophysiology *in vivo*

In this study, whole-cell or cell-attached recordings were conducted in V1 L2/3 neurons of anesthetized mice using 5–10 MΩ glass pipettes containing (in mM: potassium gluconate 125, NaCl 10, HEPES 20, Mg-ATP 3, Na-GTP 0.4, in ddH2O; 290 mOsm; pH 7.3; Alexa 488, 50 ug/ml). Signals were amplified with a Multiclamp 700B, digitized with a Digidata 1440B, using pClamp software (Molecular Devices) and stored on a PC (Dell, USA). Exposed brain region was covered with Artificial Cerebrospinal Fluid (ACSF) containing (in mM: NaCl 126, KCl 2.5, NaH2-PO4 1.25, MgCl2 1, NaHCO3 26, glucose 10, CaCl2 2.4, in ddH2O; 290 mOsm; pH 7.3). Quality of pipette was checked both electrically and optically. Pipette resistance was measured in ASCF and agarose. Pipette would be discarded if disproportional increase of resistance occurred, which indicates occlusion of pipette tip. Optically ejection of dye under 2-p imaging was also used to indicate whether the pipette was free of occlusion. During SmartACT operation, pipette resistance was sampled at 1 Hz by measuring the voltage command signal and amplifier current output during test pulses using a separate DAQ board (National Instruments, USA) and made available to other software using an HTTP server. Alternatively, the values from the MultiClamp Commander were accessed in MATLAB (Mathworks, USA) via a REST architecture. If both the electrical and optical measure agreed the tip is clogged, the pipette was retracted, discarded and a new penetration started with a new, unclogged pipette. Pipette pressure was controlled manually using a syringe or by mouth and monitored using a manometer.

For whole-cell recording, at the end of adaptive trajectory operated by SmartACT, the pipette was checked for clogging. If the pipette was clean, it was then manually advanced towards the target neuron with a low positive pressure (~20 mbar above ambient) under 2-p imaging. Pipette resistance was constantly monitored. If there was a ‘hit’, which means optically the spatial overlap between the tip and soma of the target neuron (tip contacting the plasma membrane of the neuron, causing a ‘dimple’) and electrically increase of pipette resistance were simultaneously observed, the positive pressure was released and a negative pressure was applied when holding the membrane at −70 mV to rapidly form a gigaseal. After gigaseal formation, a brief negative pressure was used to rupture the membrane inside the tip to achieve the whole-cell configuration. Membrane potential (Vm) was recorded under current clamp mode with series resistance (Rseries) appropriately compensated. We didn’t correct for liquid junction potential. Alternative, recording could be in voltage clamp mode, depending on the experiment design. For cell-attached recording, a gentle suction was applied when the tip ‘hit’ the target neuron to form a loose seal (>50 MOhm) and recording of action potentials was conducted under “I = 0” mode (current clamp without current injection).

### 2-p Targeted Single-cell Electroporation *in vivo*

**Figure 2:**
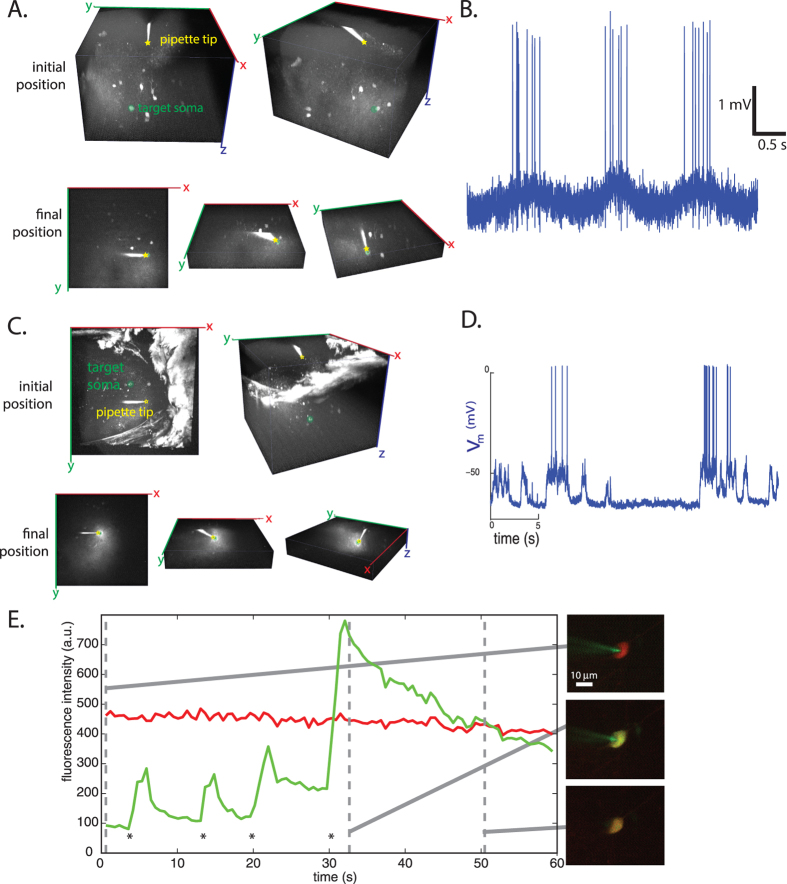
SmartACT facilitates *in vivo* experiments. **(A**,**C**) 3D visualization of pipette and labeled cells before (top) and after (bottom) adaptive approach. (**B**) Cell-attached recording from the targeted cell in (A,**D**) Whole-cell recording from the targeted cell in (**C**,**E**) (*left)*Fluorescence traces of cytosolic tdTomato (red) and Alexa 488-labeled pipette solution (green) showing four applications of the headstage electroporation protocol described in Methods (indicated by*). The fourth electroporation (t ~30 s) resulted in dye influx without loss of cytosolic tdTomato fluorescence. *(right)* 2-P-images of the tdTomato-positive neuron at the indicated time-points showing dye influx and residual tdTomato fluorescence after electroporation.

Electroporation shared many common features with the 2-p targeted cell-attached recording. Pipettes were filled with the same internal solution plus a CAG-EGFP plasmid (100–200 μg/μl, GeneScript, USA). Such experiments only required that the pipette resistance showed a slight increase while visually in contact with the cell. Electroporation in Fig. 2E. was accomplished using the Axon amplifier headstage and applying a train of 1 ms pulses of -200 nA for 1–2 seconds at 500 Hz. This protocol could be repeated if no visual confirmation of dye infiltration from the pipette into the cell was initially found. In some experiments, an Axoporator 900A (Molecular Devices USA) was used with 500 μs pulses at 50 Hz or 50 μs pulses at 500 Hz, both for 1 second duration at −12 V amplitude. No difference was observed between the results of the different electroporation methods.

## Additional Information

**How to cite this article**: Long, B. *et al.* 3D Image-Guided Automatic Pipette Positioning for Single Cell Experiments *in vivo*. *Sci. Rep.* **5**, 18426; doi: 10.1038/srep18426 (2015).

## Supplementary Material

Supplementary Information

Supplementary Movie

## Figures and Tables

**Figure 1:**
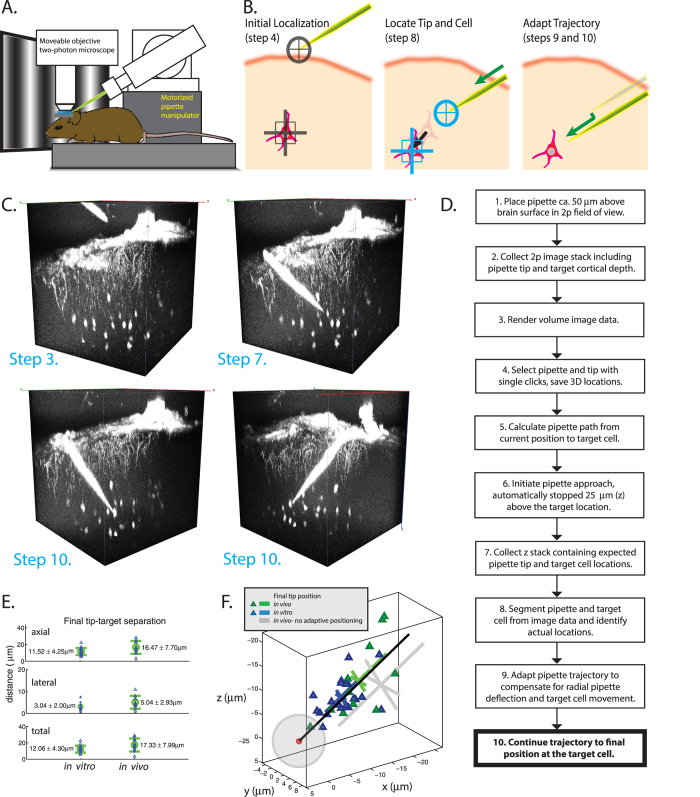
(**A**) Hardware schematic illustrating *in vivo* 2-P microscope and motorized manipulator configuration for 2-P targeted experiments (**B**) Sketch illustrating integral steps in the smartACT process. *Left*: localization of pipette tip and target. *Center*: update location of pipette and target while the pipette is deep in the brain. *Right*: Adapt trajectory and complete approach to target cell. (**C**) 3D visualization of pipette approaching target cell. The final separation between the tip and the center of the cell in this example is 6.7 microns, 6.2 microns axial (along pipette axis) and 2.5 microns lateral (perpendicular to pipette axis). (**D**) smartACT workflow. (**E**) SmartACT precisely approaches targets *in vivo* and *in vitro*: Distances between pipette tip and target cells (*in vivo*, N = 11, *right)* and target beads (*in vitro*, N = 18, *left*). (**F**) Point cloud showing distribution of final pipette positions relative to the target for smartACT approaches in *in vitro* experiments (*blue triangles*) and *in vivo* experiments (*green triangles*). The final target position is centered at the origin and the gray sphere has radius of 5 microns to approximate the soma of a pyramidal neuron, and the pipette direction is indicated in black. The cross-hairs are mean ± standard deviation along and perpendicular to the pipette axis for *in vitro* and *in vivo* smartACT approaches, as well as for N = 22 non-adaptive approaches (*grey* see Fig. S2).
