# Supplementary material for: 3D Image-Guided Automatic Pipette Positioning for Single Cell Experiments in vivo
Source: Sci Rep. 2015 Dec 22;5:18426. doi: 10.1038/srep18426 (PMC4686883; doi:10.1038/srep18426)
Supplement: Supplementary Information [file srep18426-s1.pdf]

Supplemental Information for

3D Image-Guided Automatic Pipette Positioning for Single Cell Experiments *in vivo*

Brian Long, Lu Li*, Ulf Knoblich, Hongkui Zeng, and Hanchuan Peng*

Allen Institute for Brain Science, Seattle, WA, USA.

Supplemental Movie M1 caption

Supplemental Figures S1-S6 and captions

Supplemental Movie M1.

This movie shows an annotated screencast of smartACT in use for approaching fluorescent targets. The total time for the experiment is 6m:30s including approximately 2m:30s imaging time. The video was edited for clarity, omitting imaging time, pipette approach time and some window and mouse-click operations. Targets for this *in vitro* experiment were 2.0-micron green fluorescent beads in 1.2% agarose, and the pipette internal solution was Alexa 594.

Supplemental Figures S1-S6

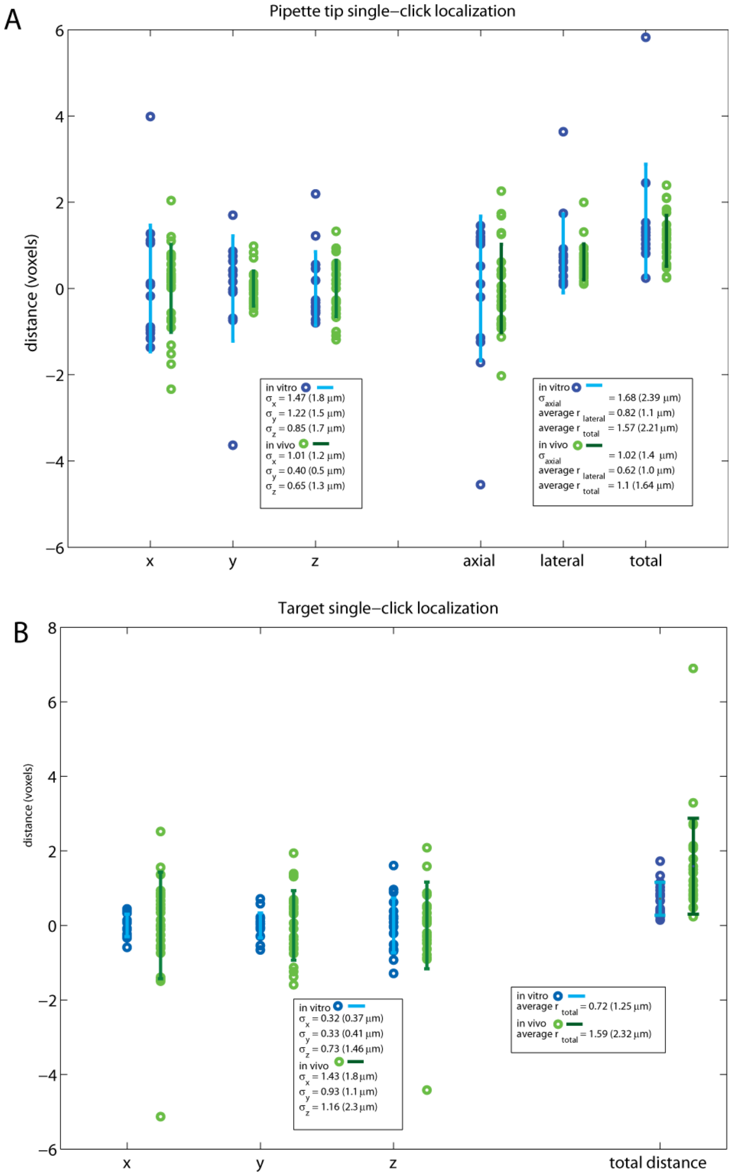

Figure S1. Precision of virtual-finger single-click localization of pipette tip and cell positions. N=13, 15 localizations of the labeled target cell in each of two experiments *in vivo* (green). N = 11, 14 localizations of the pipette tip above the surface of the brain in the same image stacks (green). N = 18 localizations of a 2 micron fluorescent bead in agarose (*in vitro*) (blue), N = 14 localizations of the pipette tip in the same image stack (blue). All distances are from the mean position of the respective pipette or target localizations.

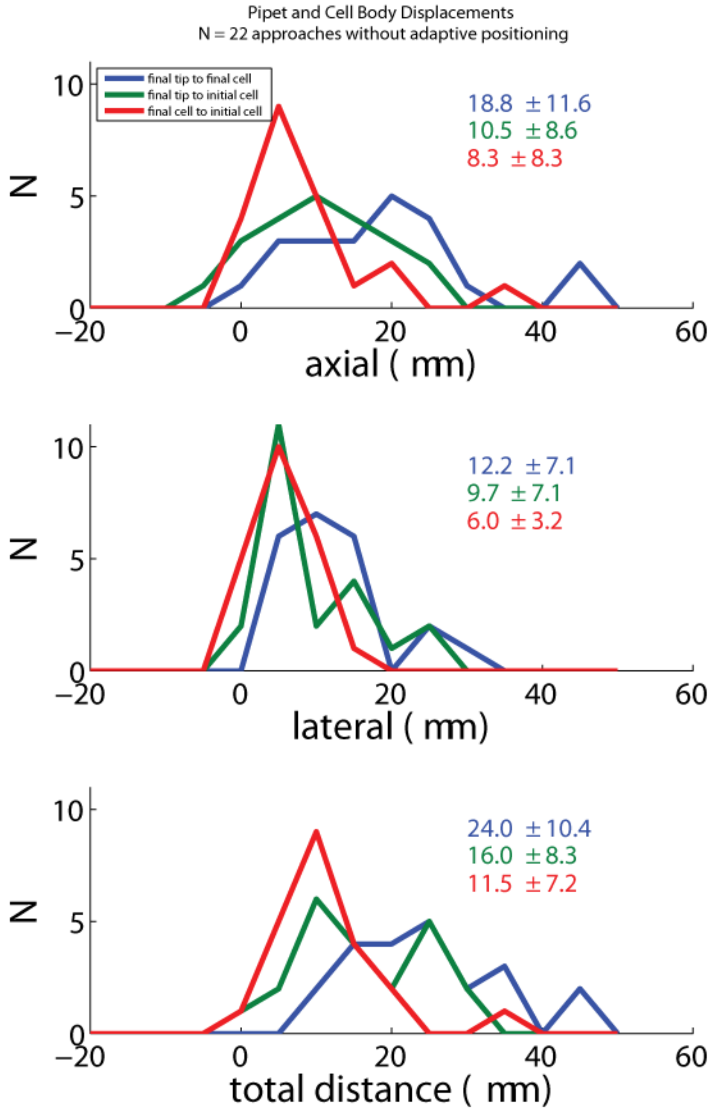

Figure S2 Histograms of pipette tip to cell center distances without image-guided adaptive pipette movement. Histograms of distance along the pipette axis (parallel), lateral to the pipette axis (perpendicular) and total distance for N = 22 non-adaptive approaches *in vivo*. The distance between the final target position and the initial target position (red) quantifies cell body displacement during the approach. The distance between the final pipette tip position and the initial target position (green) quantifies pipette tip deflection during the approach. The distance between the final target position and the final pipette tip position (blue) quantifies the final separation at the terminus of the non-adaptive approach. The intended final position is 10-12 microns axial and 0 microns lateral from the center of the cell body. Mean and standard deviations are color-coded with their respective histogram line plots. Automation of pipette targeting *in vivo* without adaptive corrections yields substantial variation in final pipette-target separation (blue) due to errors in both pipette displacement (green) and target cell movement (red).

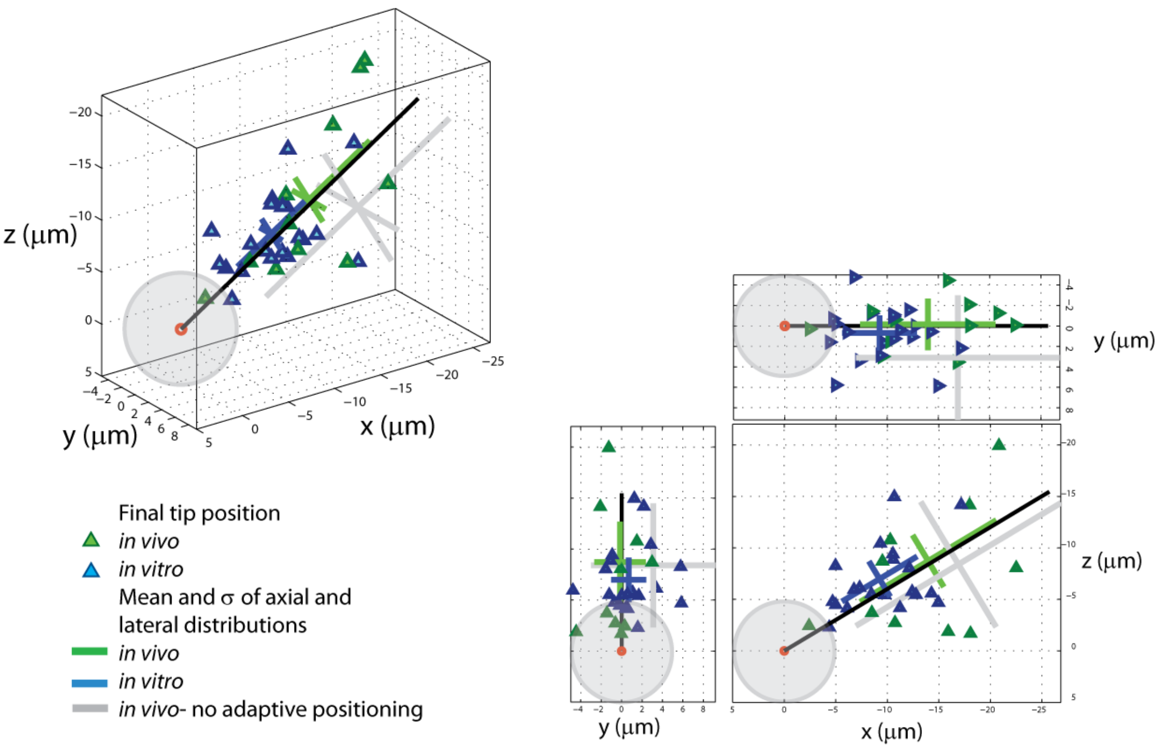

Figure S3. Final locations of pipette tip relative to final target positions across experimental methods (additional views of data in Fig. 1 F). Adaptive corrections *in vitro* and *in vivo* (blue and green, respectively) lead to reduced variability in final pipette position, as compared to non-adaptive approaches (grey) The final target position is centered at the origin and the gray sphere has radius of 5 microns to approximate the soma of a pyramidal neuron. The cross-hairs are centered on the mean coordinates and crosses indicate standard deviation along and perpendicular to the pipette axis for *in vitro* and *in vivo* smartACT approaches, as well as for N = 22 non-adaptive approaches (*grey*).

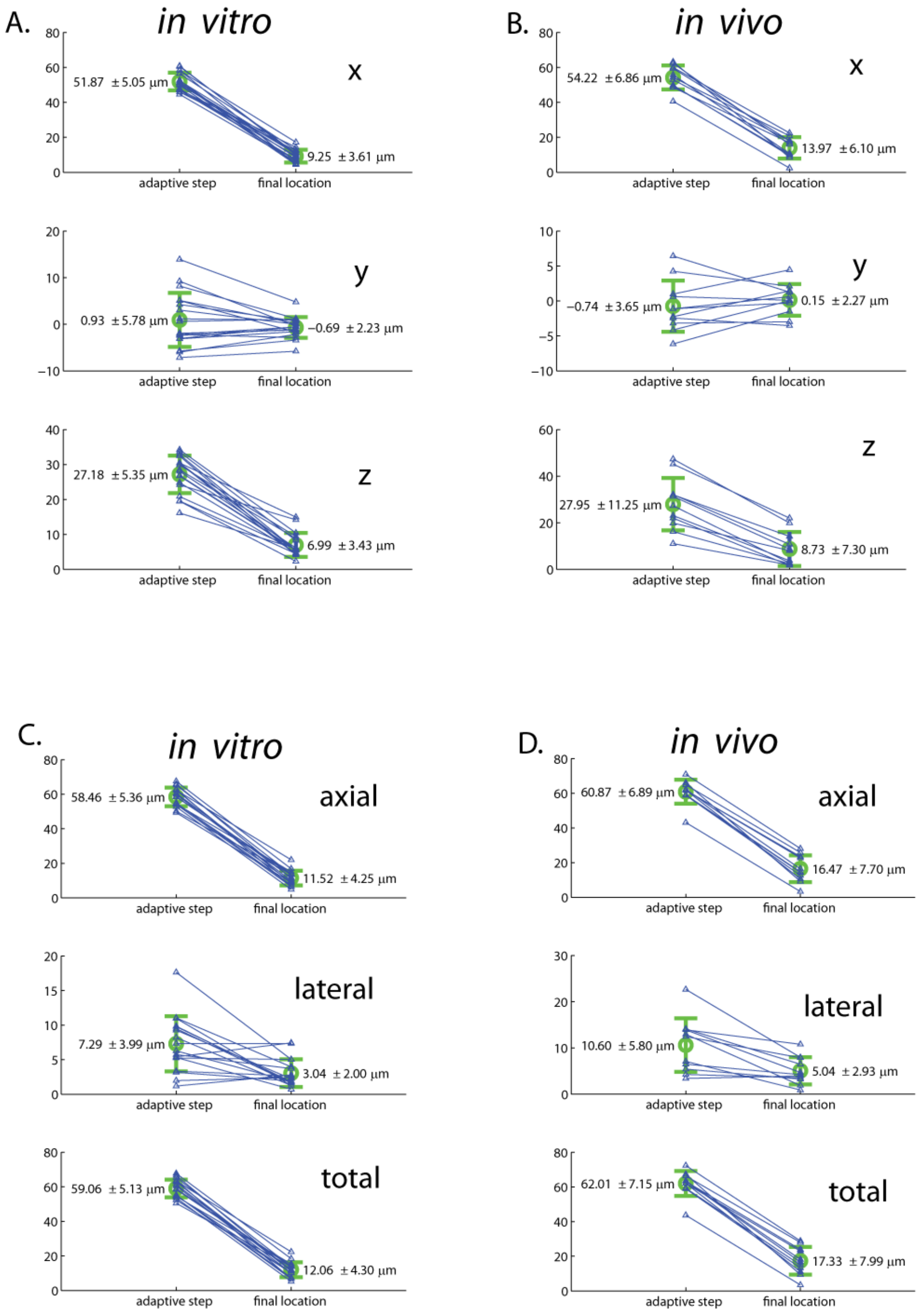

Figure S4. Adaptive corrections *in vivo* (N = 11) and *in vitro* (N = 18) reduce variability in pipette location. Cartesian coordinates (A and B) and pipette-resolved components (C and D) of the vector between the target position and the pipette tip *in vitro* (A and C) and *in vivo* (B and D). Each plot shows the distances at the adaptive step (step 8 in Fig. 1 B) and at the final location (step 10 in Fig. 1. B), illustrating that the adaptive correction improves lateral distance by ~2x by (C and D, middle panels).

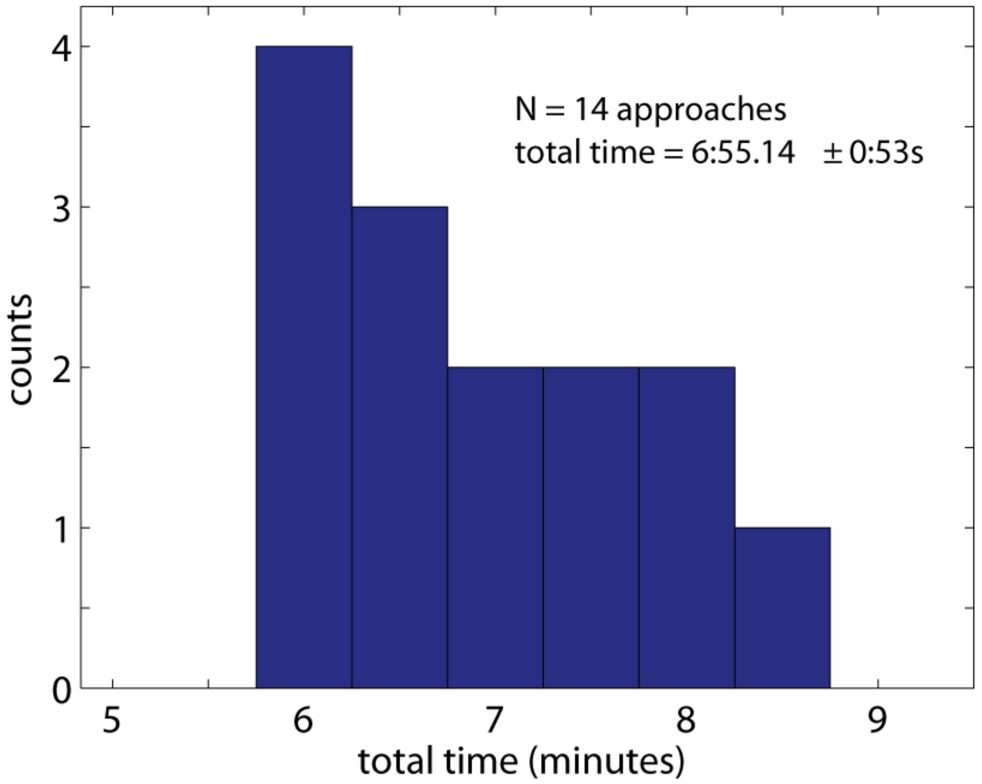

FIGURE S5 Total time for adaptive pipette approaches. The total time for N = 14 adaptive approaches, including approximately 2.5 minutes for image stack collection as discussed in the text.

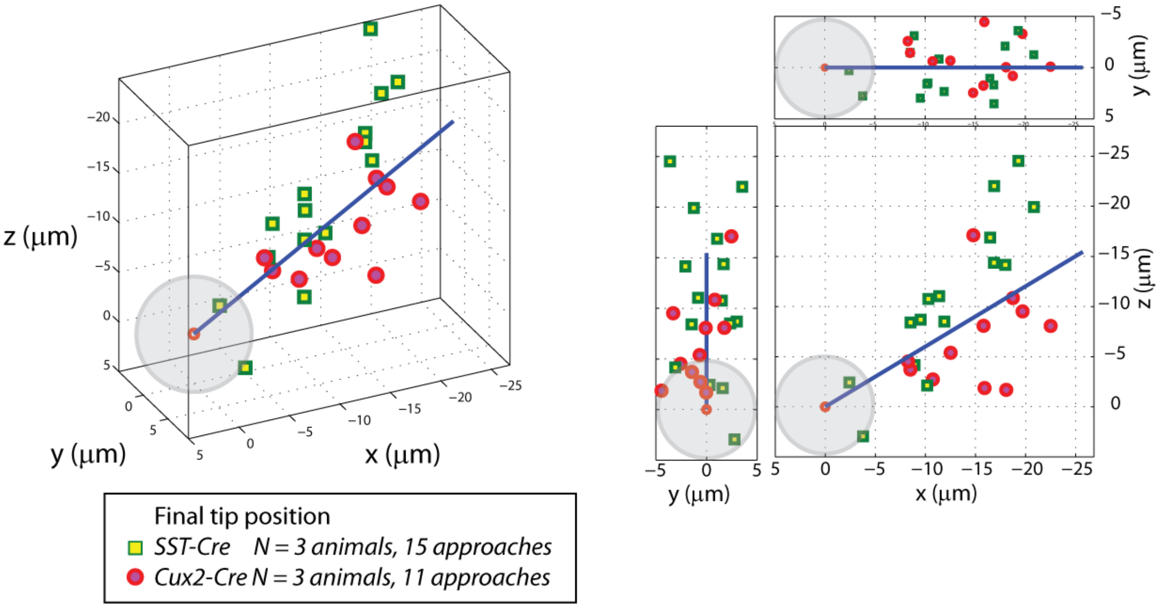

Figure S6 Final locations of pipette tip relative to final target positions for Cux2-Cre and SST-Cre mice. These data include all of the *in vivo* smartACT approaches analyzed in this study, both for methods characterization and for electrophysiology. The target cell-type is indicated by marker color (see legend).
